# Association between store food environment and customer purchases in small grocery stores, gas-marts, pharmacies and dollar stores

**DOI:** 10.1186/s12966-017-0531-x

**Published:** 2017-06-05

**Authors:** Caitlin E. Caspi, Kathleen Lenk, Jennifer E. Pelletier, Timothy L. Barnes, Lisa Harnack, Darin J. Erickson, Melissa N. Laska

**Affiliations:** 1 Department of Family Medicine and Community Health, Program in Health Disparities Research, University of Minnesota, 717 Delaware St. SE, Minneapolis, MN 55414 USA; 2 Division of Epidemiology and Community Health, Suite 300, University of Minnesota, 1300 South 2nd St, Minneapolis, MN 55454 USA; 3 Statewide Health Improvement Program, Minnesota Department of Health, Saint Paul, MN 55164 USA

**Keywords:** Community nutrition, Customer purchases, Healthy Eating Index, Corner stores

## Abstract

**Background:**

Purchases at small/non-traditional food stores tend to have poor nutritional quality, and have been associated with poor health outcomes, including increased obesity risk The purpose of this study was to examine whether customers who shop at small/non-traditional food stores with more health promoting features make healthier purchases.

**Methods:**

In a cross-sectional design, data collectors assessed store features in a sample of 99 small and non-traditional food stores not participating in the Special Supplemental Nutrition Program for Women, Infants, and Children (WIC) in Minneapolis/St. Paul, MN in 2014. Customer intercept interviews (*n* = 594) collected purchase data from a bag check and demographics from a survey. Store measures included fruit/vegetable and whole grain availability, an overall Healthy Food Supply Score (HFSS), healthy food advertisements and in-store placement, and shelf space of key items. Customer nutritional measures were analyzed using Nutrient Databases System for Research (NDSR), and included the purchase of ≥1 serving of fruits/vegetables; ≥1 serving of whole grains; and overall Healthy Eating Index-2010 (HEI-2010) score for foods/beverages purchased. Associations between store and customer measures were estimated in multilevel linear and logistic regression models, controlling for customer characteristics and store type.

**Results:**

Few customers purchased fruits and vegetables (8%) or whole grains (8%). In fully adjusted models, purchase HEI-2010 scores were associated with fruit/vegetable shelf space (*p* = 0.002) and the ratio of shelf space devoted to healthy vs. less healthy items (*p* = 0.0002). Offering ≥14 varieties of fruit/vegetables was associated with produce purchases (OR 3.9, 95% CI 1.2–12.3), as was having produce visible from the store entrance (OR 2.3 95% CI 1.0 to 5.8), but whole grain availability measures were not associated with whole grain purchases.

**Conclusions:**

Strategies addressing both customer demand and the availability of healthy food may be necessary to improve customer purchases.

**Trial registration:**

ClinialTrials.gov: NCT02774330. Registered May 4, 2016 (retrospectively registered).

**Electronic supplementary material:**

The online version of this article (doi:10.1186/s12966-017-0531-x) contains supplementary material, which is available to authorized users.

## Background

Access to high-quality, nutritious food has long been recognized as a contributor to individual dietary behaviors [1–7]. In the U.S., urban residents purchase food and beverages at an array of businesses besides supermarkets [5, 7–11], including small grocery stores (corner stores, bodegas) [10, 12–18], as well as other limited-service businesses, like gas-marts, dollar stores, and pharmacies [19–24]. Together these have been referred to as “small and non-traditional” urban food retailers [25]. These stores attract loyal customers who make food and beverage purchases daily or multiple times a week [12–14, 17, 18, 26]. Small and non-traditional food stores are common in high-minority and low-income neighborhoods, which are also less likely to have supermarkets [27–32].

Small and non-traditional stores offer an abundance of less nutritious foods like sugar-sweetened beverages, salty snacks, and candy [27, 33–37]. Consequently, purchases at small and non-traditional food stores tend to have poor nutritional quality [13–17, 25, 26, 30, 38], and have been associated with poor health outcomes, including increased obesity risk [39–41].

Offering nutritious food at small and non-traditional food stores may result in healthier purchases [18, 26]. In a recent multilevel study in New York City by Ruff et al. (2016), customers at stores that offered more varieties of fresh fruits and vegetables were more likely to purchase produce and less likely to purchase sugar-sweetened beverages than customers at stores that offered fewer varieties of fresh fruits and vegetables [26]. A study by Martin et al. (2012) in small food stores in Hartford, CT showed that each additional type of produce available at the store was associated with 12–15% higher odds of purchasing a fruit or vegetable [18]. While the majority of such stores carry some fresh produce and staple foods [18, 22, 23, 26, 37, 42], purchases of these items are uncommon [16, 25–27, 43]. Indeed, small food store owners cite customer demand as one of the primary considerations in their decision to stocking health food, and often perceive demand to be low [16, 44]. Some features of the food environment have the potential to influence customer decision-making and boost demand, such as store marketing features like advertising, product promotions, and in-store placement of nutritious foods [44–46].

Yet, marketing strategies to encourage the purchase of nutritious foods have only been evaluated to a limited extent in small and non-traditional stores [47–49].

Overall, it is difficult to discern from the public health literature which health-promoting store characteristics are most likely to encourage healthy behavior [45, 50]. In most interventions, marketing strategies are used in combination, making it difficult to identify which ones are effective [28, 45]. Furthermore, evaluations of interventions have often stopped short of using objective purchase data or incorporating rigorous measures of store marketing [17, 28, 42, 51, 52].

The purpose of this study is to examine whether customers who shop at small and non-traditional food stores with more health-promoting features (i.e., marketing, overall healthy food supply, more fruits, vegetables and whole grains) have healthier purchases (i.e., more fruits and vegetables, more whole grains, and higher Healthy Eating Index scores), using objective data on store features and customer purchases. Understanding which health-promoting features are associated with customer purchases in small stores may be useful in designing interventions to improve the nutritional quality of food purchases at small stores [53].

## Methods

### Design

The study took place between July and November 2014 as part of the baseline evaluation of a city ordinance regulating minimum stocking requirements for food retailers in Minneapolis, MN, USA, as well as comparable stores in St. Paul, MN, USA (the control site). The Minneapolis Staple Foods Ordinance [54], effective since April 2015, requires all licensed grocery stores in Minneapolis to meet specific stocking requirements for 10 staple food categories [54], including fruits and vegetables, whole grains, and low-fat dairy. The Minneapolis Staple Foods Ordinance included most small and non-traditional food stores. All data in the current study draw from pre-policy assessments, though the study size was based on the sample size of stores and customers needed to detect a change in store environment and customer purchases over time.

In teams of two, data collectors visited each store to assess the store environment (during one visit) and to collect purchase data from customers exiting the store (during at least one other visit). Data collection took place between 9:00 am and 6:00 pm Monday through Friday, and between 11:00 am and 7:00 pm Saturday and Sunday. Data collectors asked permission from a store employee prior to collecting data, and obtained informed consent before conducting customer interviews. The study was approved by the Institutional Review Board at the University of Minnesota.

### Sample

#### Store sample, selection, and enrollment

Store lists were obtained from relevant licensing agencies (i.e., the Minneapolis Health Department for Minneapolis stores and the Minnesota Department of Agriculture for St. Paul stores). Stores were exempt from the ordinance if they would not reasonably be expected to stock a minimal amount and variety of foods (e.g., produce stands), as well as liquor stores and specialty stores (e.g., spice shops). Stores located in the core downtown commercial district were also exempt based on the ordinance. Because the evaluation targeted ordinance-eligible retailers that were not already expected to meet ordinance requirements, stores were excluded if they were supermarkets, mass-merchandizers, or listed in the state Special Supplemental Nutrition Program for Women, Infants, and Children (WIC) database, (and therefore already meeting specific program requirements for stocking healthy, staple foods). Finally, we excluded stores that had invalid licensing addresses.

Out of 255 eligible stores, 180 were randomly selected to participate in the study. After visiting these stores in person, 20 were identified as ineligible due to new participation in WIC (*n*= 5), misclassification of supermarkets and/or exempt stores on license listings (*n* = 10), and going out of business by the time of our store visit (*n* = 5). Three more agreed first to have customer intercept data collected at the store, but soon after lost their grocery license or otherwise became exempt from the ordinance prior to the store assessment data collection. An additional 17 refused or did not give active consent to participate in the store assessment (11 stores stated that corporate policy precluded them from giving consent, 5 stores did not give a reason, and in 1 store the manager was not able to be reached). A final sample of 140 stores participated in the study store assessment (described in detail below) at baseline.

#### Customer sample, selection, and recruitment

Data collectors were paired based on their availability and visited each store in teams of two. The majority of data collectors were White and female. Upon visiting the store, they introduced themselves as members of a research team at the University of Minnesota and described the research as a study about the healthfulness of customer purchases.

Data collectors stood outside participating stores and attempted to recruit each customer who exited the store with a bag or visible food/beverage purchase. Data collectors carried out recruitment for 30–60 min per visit. If at least 1 survey was completed during the first 30 min of a visit, data collectors stayed an additional 15–30 min. The recruitment target at each store was 5 participants. If this target was not met during the first visit to the store, additional visits were made in an attempt to reach the target. Participants were eligible if they were English-speakers, at least 18 years old, and had made a food/beverage purchase at the store. A $10 incentive was offered to encourage participation. Data collectors performed a bag check in which they recorded details about each food and beverage purchased by the participant, including the product name (e.g., Flaming Hot Cheetos), amount/size (e.g., 3.5 oz. bag), quantity (e.g., 1 each), and price paid (e.g., $1.29). In addition, participants completed a brief survey that included shopping behaviors, perceptions of the local food environment, and demographics (age, gender, race/ethnicity, education, employment). A total of 668 customers were recruited, representing a diverse sample of Minneapolis/St. Paul residents. The overall participation rate was 35%. Based on nonparticipation data, customers who were identified as Black were more likely to participate than those who were identified as White, but there were no systematic differences in participation by gender. Details on recruitment, eligibility status, and nonparticipation have been previously published [55] and are detailed futher in Additional file 1.

### Measures

#### Store environment

The store environment was assessed using a tool modified from the Yale Rudd Center [56], which is similar in structure to the validated Nutrition Environment Measure Survey in Stores (NEMS-S) instrument in that they both record availability, price, and (for fresh fruits and vegetables) quality. The Rudd Center tool focuses on WIC-approved items, and in the current study, the tool was further adapted to align with Minneapolis Staple Food Ordinance requirements. A total of 69 food items were evaluated [22, 23]. Agreement for all 69 items was above 80% in inter-rater reliability analyses.

Fruit and vegetable availability at the store included two measures: (1) pounds of fresh fruits and vegetables (with no other ingredients added), for which standard item weights (with refuse weight added) [57, 58] were multiplied by the count of items; and (2) the number of varieties of fresh, frozen and canned fruits and vegetables (with no other ingredients added, except for salt in canned vegetables). Store-level whole grain measures included the number of pounds and varieties of: (1) whole grain–rich cereals, bread, and tortillas; (2) brown rice; and (3) other whole grains and whole grain-rich products products (e.g., plain uncooked oats, whole cornmeal, unpopped popcorn, whole-wheat flour, teff flour, quinoa, pasta). Whole grain-rich snacks (e.g. tortilla chips, pre-popped popcorn) were not included in the whole grain measure. Data collectors counted items as whole grain-rich if the first ingredient listed on the package was a whole grain [53].

Data were used to create a Healthy Food Supply Score (HFSS) score for each store, which summarized availability, price, quality, and variety of inventory [56]. The summary score had a possible range of 0–31. Higher scores indicate greater availability of healthier foods at the store. The score has previously been used to evaluate overall healthfulness in both WIC and non-WIC stores [22, 56].

Store marketing features, including food advertisements and product placement, were also assessed at each store. Measures were based on a modified tool developed by the CX^3^ retail scoring system [59], adapted in the current study [60]. Data collectors recorded whether there were any images of “healthy” foods (e.g., fruits and vegetables, whole grains, beans, nuts and seeds) on store exteriors (storefront doors or windows) or the interiors (next to, below, or on the floor standing to the checkout, or hanging from the store’s ceiling). Stores were then characterized according to whether they had healthy images present on the interior (yes/no) and exterior (yes/no).

Product placement measures assessed whether fresh fruits and vegetables could be seen from the front entrance (yes/no), or if any healthy food items (e.g., bagged nuts/seeds with no added sugar, fresh fruit) were within reach of the cash register (yes/no). Inter-rater agreement ranged from 88% to 100% for store marketing features.

Shelf space for fruits, vegetables, less healthful non-alcoholic beverages, and salty snacks was measured with standard tape measures in inches and rounded to the nearest foot. Less healthful beverages included all non-alcoholic beverages except plain (unflavored) water, unsweetened milk, and 100% juice. Salty snacks included chips, popped and/or flavored popcorn, salted meat snacks, and similar salty, processed foods. It did not include nuts and seeds, rice cakes, or plain crackers. Inter-rater agreement was within 15% for shelf space measures and ranged from 72% (salty snacks) to 89% (vegetables).

#### Customer purchases

Food and beverage purchase data were entered into the nutrient analysis program Nutrition Data System for Research (NDSR). NDSR University of Minnesota Nutrition Coordinating Center Food and Nutrient Database serves as the source of food composition information in NDSR [61]; the database includes over 18,000 foods including 8000 brand name products. The USDA Nutrient Data Laboratory is the primary source of nutrient values and nutrient composition [62]. NDSR generates food servings for 168 food subcategories in 9 major food groups. If the total number of fruit and vegetable servings purchased (excluding fruit/vegetables juices and fried vegetables) was at least 1, customers were characterized as having made a fruit or vegetable purchase. If the total number of servings of whole grain-rich foods purchased was at least 1, customers were characterized as having made a whole grain purchase. Due to the way in which NDSR classifies foods, it was not possible to completely align the fruit/vegetable and whole grain-rich food measures in the store environment assessment with calculation of these food categories in NDSR. For example, the store environment measure does not include vegetables in mixed dishes such as frozen dinners, whereas vegetables from all food sources are included in the NDSR derived estimate.

To evaluate the nutritional quality of food purchases in a global way, a Healthy Eating Index-2010 (HEI-2010) score was created for each purchase using methods publically at the National Institutes of Health (NIH) [63]. The HEI-2010 is a US Department of Agriculture (USDA) tool used to measure the degree to which a diet or food source is consistent with federal dietary guidelines [64]. HEI-2010 has a range of 0–100, with higher scores indicating better alignment with recommendations [65]. HEI scores are computed by deriving ratios of dietary constituents to energy, and then scoring each of the 12 subcomponents according to minimum and maximum standards outlined by the USDA [65]. The HEI can be applied across different levels of the food supply chain and, regardless of the level, the scores are comparable [66]. It is a valid measure for assessing nutritional quality of the food environment [67–70] or food purchases [71].

### Statistical analyses

The analysis included stores at which both data on store and customer purchases were collected. Of the 140 stores in which store environment data were collected, 18 additional stores were excluded from the analysis because they did not give active consent for the recruitment of customers for interviews, and at 22 stores, no customer was successfully recruited for an interview. Recruitment was more challenging at stores where customers may have been stopping for a brief errand, or where many customers purchased only non-food items, such as gas-marts and pharmacies. The remaining 100 stores were categorized as corner stores, gas-marts, dollar stores, pharmacies, and one general retailer. The general retailer was excluded from this analysis because it could not be included in models controlling for store type. Thus, the final analytic sample was comprised 99 stores at which 594 customer intercept interviews took place. The majority of stores (56%) had 5 to 10 customers recruited and 10% of stores had more than 10 customers recruited, with a maximum of 20 customers.

We calculated descriptive statistics for all measures. For normally distributed continuous measures we calculated means with standard deviations; for continuous measures that were not normally distributed we calculated medians with the first and third quartile. For dichotomous measures we calculated percentages. To examine the associations between store environment features and purchase outcomes, we computed multilevel linear regression models (the for HEI-2010 outcome) or logistic regression models (for the fruit/vegetable and whole grains purchase outcomes), with store identification treated as a random effect to account for nesting of customers within stores. We computed a series of three models for each independent variable—(1) unadjusted; (2) adjusted for individual covariates (age, gender, race/ethnicity, and education); (3) adjusted for individual covariates and store type. The number of store aisles and registers were not included in our final models because they were highly correlated with each other and with store type. In linear models, we calculated least square means of HEI-2010 for each level for categorical independent variable; for the continuous HFSS variable, we present the regression coefficients and standard errors. For interpretability, the mean HEI-2010 at the median and 25th and 75th percentiles of HFSS were also calculated. Availability measures for fruits and vegetables and whole grain-rich foods were treated as categorical variables, approximating tertiles or quartiles based on each variable’s distribution. In logistic models, odds ratios with 95% confidence intervals used the lowest level of the independent measure as the reference group, unless the lowest group was “none,” in which case the second lowest group was used as the reference group and estimates were not provided for the “none” category [26]. Analyses were done using SAS/STAT software Version 9.4 (SAS Institute, 2002–2012); the significance level was *p* < 0.05.

## Results

### Participant characteristics and purchases

Table 1 presents participant characteristics and purchases. Eight percent of participants purchased ≥1 serving of fruits and vegetables. Eight percent also purchased ≥1 serving of whole grain-rich foods, three-quarters of whom purchased a whole grain-rich snack food (e.g., tortilla chips, flavored popcorn). The average HEI-2010 score was 31. Participants were on average 40 years old; 58% were male. The sample was diverse in terms of race/ethnicity and education, and employment.

**Table 1:** Participant and small and non-traditional food store characteristics^a^

Participant characteristics (*n* = 594)	
*Dependent variables*	*N* (%)
Purchase of a fruit or vegetable (≥1 serving)	49 (8)
Purchase of a whole grain (≥1 serving)	50 (8)
	Mean (SD)
HEI-2010^b^ of purchase	31 (13)
*Covariates*	
Age	40 (15)
	*N* (%)
Gender (male)	341 (58)
Race/ethnicity	
Hispanic	19 (3)
White Non-Hispanic	285 (48)
Black Non-Hispanic	214 (36)
Native American	12 (2)
Asian	22 (4)
Other	20 (3)
Multi-race	19 (3)
Education	
High school or less	214 (36)
Some college	220 (37)
College degree	158 (27)
Employment	
Employed	381 (64)
Unemployed	149 (25)
Other (student, retired, disability)	63 (11)
Store characteristics (*n* = 99)	
*Independent variables*	Median (Q1, Q3)
Pounds of fruits/vegetables (fresh and frozen)	15 (0, 52)
Varieties of fruits/vegetables (fresh, frozen and canned)	9 (5, 15)
Pounds of whole grains	14 (2, 37)
Varieties of whole grains	2 (1, 3)
Shelf space (in feet)	
Fresh fruit/vegetables	4 (0, 9)
Unhealthy foods (snacks & sugar-sweetened beverages)	359 (244, 420)
Ratio of fresh fruits/vegetables to unhealthy foods	0.01 (0, 0.03)
	Mean (SD)
Healthy Food Supply Score (HFSS)^c^	11 (5)
	*N* (%)
Healthy impulse buys at checkout	62 (63)
Fruit/vegetable impulse buys at checkout	20 (20)
Healthy ads/promos: exterior	39 (39)
Healthy ads/promos: interior	22 (22)
Fruit/vegetables visible from entrance	35 (35)
*Covariates*	*N* (%)
Store type	
Corner/small grocery	40 (40)
Gas/food mart	30 (30)
Dollar store	8 (8)
Pharmacy	21 (21)
Number of aisles	
0–4	30 (31)
5–8	35 (36)
9+	32 (33)
Number of registers	
1	35 (36)
2–3	39 (40)
4_+_	23 (24)

*Q1* first quartile, *Q3* third quartile, *SD* standard deviation

^a^Data collected in Minneapolis/St. Paul, MN in 2014

^b^Possible range 0–100

^c^Possible range 0–31

### Store environment characteristics

As presented in Table 1, store sample was comprised of 40% corner stores, 30% gas-marts, 8% dollar stores, and 21% pharmacies. Stores carried a median of 15 lb of fresh and frozen fruits and vegetables, and a median of 9 varieties of fresh, frozen, and canned fruits and vegetables. Stores had a median of 14 lb of whole grains available in 2 varieties. The overall Healthy Food Supply Score (HFSS) was 11. Stores had a median of 4 ft of shelf space for fresh fruits and vegetables, compared with a median of 359 ft for salty snacks and sugar-sweetened beverages. Nearly two-thirds of stores had healthy impulse buys at the point-of-purchase, but a minority of stores had healthy interior or exterior advertisements or produce in visible areas.

Table 2 presents the association between the overall store environment (healthy advertisements, impulse buys, shelf space, and overall healthy food availability) and the overall nutritional quality (HEI-2010) of purchases at that store. Controlling for individual characteristics, HEI-2010 scores for purchases were higher in stores with greater shelf space for fruits and vegetables (*p* = 0.0006), a higher ratio of shelf space for healthy vs. less healthy food (*p* < 0.0001), and higher overall HFSS (*p* = 0.03). Healthy advertisements on the store exterior (*p* = 0.03) were unexpectedly associated with lower HEI-2010 scores. After controlling for store type, shelf space measures remained statistically significant, while exterior advertisements and HFSS were not. Neither the presence of healthy impulse buys, nor the presence of interior healthy advertisements were associated with purchase HEI-2010 scores.

**Table 2:** Association between overall healthy food availability and promotions and Healthy Eating Index (HEI-2010) of purchases^a^

	HEI-2010					
	Model 1^b^		Model 2^c^		Model 3^d^	
	LS means (HEI-2010)	*p*	LS means (HEI-2010)	*p*	LS means (HEI-2010)	*p*
Healthy impulse buys		.43		.45		.51
Yes	31.3		31.4		31.4	
No	30.3		30.4		30.6	
Healthy advertisements: exterior		.04		.03		.20
Yes	29.5		29.4		30.0	
No	32.0		32.2		31.8	
Healthy advertisements: interior		.90		.67		.79
Yes	30.8		30.6		31.4	
No	31.0		31.2		31.0	
Shelf space: Fresh fruits/vegetables (in feet)		.0004		.0006		.002
None	29.9		29.7		28.8	
Low (1–5)	28.5		28.8		30.8	
Higher (>5)	34.3		34.3		35.2	
Shelf space ratio: Fruits/vegetables to unhealthy		<.0001		<.0001		.0002
None	29.8		29.5		28.8	
Low (.002–014)	28.4		28.5		30.2	
Higher (>.014)	35.0		35.1		35.9	
Healthy Food Supply Score (HFSS)^e^	(β = 0.3; se = 0.1)	.02	(β = 0.3; se = 0.1)	.03	(β = 0.2; se = 0.2)	0.2
8	29.8		29.7		29.5	
9.5	30.1		30.1		30.2	
11.5	31.0		30.8		30.7	

^a^Data collected in Minneapolis/St. Paul, MN in 2014

^b^unadjusted

^c^adjusted for age, gender, race/ethnicity, education

^d^adjusted for age, gender, race/ethnicity, education and store type

^e^HFSS modeled as continuous measure (β = regression coefficient; se = standard error); predicted means of HEI (range 0–100) are shown for the Q1 (8), median (9.5), and Q3 (11.5) values of HFSS (range 1–31) only for interpretability purposes

Table 3 presents the associations between store availability and purchasing at least one serving of fruit or vegetables. Controlling for individual characteristics, customers had 3 times greater odds of purchasing at least one serving of fruits and vegetables if the store carried at least 90 lbs. of fresh or frozen fruits and vegetables, compared with stores that sold less than 30 lbs. (95% CI 1.2 to 7.6). Customers also had 2.8 times greater odds of purchasing at least one serving of fruits and vegetables if the store stocked more than 14 varieties of fresh, frozen, and canned fruits or vegetables, compared to stores that sold less than 7 varieties (95% CI 1.1 to 7.0). Higher shelf space of fruits and vegetables (OR 2.6, 95% CI: 1.0–6.5) and a higher healthy-to-less-healthy shelf space ratio (OR 3.0, 95% CI: 1.2 to 7.3) were both associated with greater odds of purchasing at least one serving of fruits and vegetables. When controlling for store type, only the varieties of fresh, frozen and canned fruits and vegetables (OR 3.9, 95% CI 1.2 to 12.3) remained statistically significant. Having produce visible from the entrance was also statistically significant (OR 2.3 95% CI 1.0 to 5.8).

**Table 3:** Association between fruits/vegetables and whole grains availability and purchases of fruits/vegetables and whole grains^a^

	Model 1^b^		Model 2^c^		Model 3^d^	
	OR	(95% CI)	OR	(95% CI)	OR	(95% CI)
Fruits/Vegetables:	Odds of fruits/vegetables purchased^d^					
Pounds: fresh & frozen						
None	--	--	--	--	--	--
1–29	ref		ref		ref	
30–89	1.2	(0.4, 3.8)	1.2	(0.4, 3.9)	1.1	(0.3, 3.9)
≥ 90	3.6	(1.5, 8.9)	3.0	(1.2, 7.6)	3.0	(0.9, 9.9)
Varieties: fresh, frozen, & canned						
0–6	ref		ref		ref	
7–13	1.7	(0.7, 4.2)	1.7	(0.6, 4.5)	1.8	(0.6, 5.1)
≥ 14	2.9	(1.2, 7.2)	2.8	(1.1, 7.0)	3.9	(1.2, 12.3)
Healthy impulse buys						
Yes	1.2	(0.6, 2.6)	1.3	(0.6, 2.8)	1.3	(0.6, 2.9)
No	ref		ref		ref	
Shelf space: fresh						
None	--	--	--	--	--	--
Low	ref		ref		ref	
Higher	3.2	(1.3, 8.2)	2.6	(1.0, 6.5)	2.1	(0.7, 5.8)
Shelf space ratio						
None	--	--	--	--	--	--
Low	ref		ref		ref	
Higher	3.0	(1.3, 7.4)	3.0	(1.2, 7.3)	2.7	(0.9, 7.9)
Visible from entrance						
Yes	2.0	(1.0, 4.1)	1.9	(0.9, 4.0)	2.3	(1.0, 5.8)
No	ref		ref		ref	
Whole Grains:	Odds of whole grains purchase^e^					
Pounds						
0–4	ref		ref		ref	
5–14	1.7	(0.8, 3.7)	1.6	(0.7, 3.4)	1.9	(0.7, 5.1)
15–29	0.7	(0.2, 2.0)	0.7	(0.2, 2.1)	1.2	(0.3, 4.5)
≥ 30	1.7	(0.8, 3.6)	1.5	(0.7, 3.4)	1.8	(0.5, 6.0)
Varieties						
0–1	ref		ref		ref	
2	0.8	(0.3, 1.9)	0.7	(0.3, 1.8)	0.8	(0.2, 2.7)
3	0.7	(0.3, 1.6)	0.6	(0.3, 1.5)	0.5	(0.1, 1.6)
≥ 4	1.5	(0.7, 3.2)	1.4	(0.7, 3.1)	1.1	(0.3, 3.8)

*OR* odds ratio, *CI* confidence interval, *Ref* referent group

^a^Data collected in Minneapolis/St. Paul, MN in 2014

^b^unadjusted

^c^adjusted for age, gender, race/ethnicity, education

^d^adjusted for age, gender, race/ethnicity, education and store type

^e^ ≥ 1 serving

Neither the number of pounds nor the number of varieties of whole grains was associated with whole grain purchases.

## Discussion

In Minneapolis and St. Paul, MN, a number of store availability and marketing features were associated with more healthful customer purchases in corner stores, gas-marts, dollar stores, and pharmacies. In general, healthy purchases were more consistently associated with healthy food availability measures (including pounds, varieties, shelf space for fruits and vegetables, and overall healthy food supply) than with healthy food marketing features. These findings are largely consistent with a recent study of New York City bodegas by Ruff et al. (2016), which demonstrated that carrying fewer varieties of produce was significantly associated with a lower odds of purchasing produce ﻿among customers﻿, but that healthy product promotions were not related purchases [26].

Recent recommendations from an expert panel convened by the Robert Wood Johnson Foundation’s Healthy Eating Research (HER) program have included suggested standards for the availability and marketing of healthy foods in small food stores [53]. These recommendations suggest that stores stock at least 10 (at a “basic” level), and up to 14 (at a “preferred” level) varieties of fruits and vegetables. In the current study, customers had about 3 times greater odds of purchasing produce at stores that offered at least 14 varieties, although longitudinal assessments will be necessary to determine the actual impact of stocking changes on purchasing behavior.

There was no relationship between the availability of whole grains and whole grain purchases in the current study. It may be that single-serving items (including fruit) are a more relevant target for promotions in our store sample than items like whole grain-rich bread and cereal. Most stores did, however, carry whole grain-rich items in quantities that approach HER recommendations for whole grains [53].

Marketing features promoting healthy purchases were not consistently associated with healthy food purchases. In-store healthy food promotions did not appear to be relevant to customer behavior, perhaps because of the pervasiveness of less healthy marketing on the store interior and exterior. Indeed, a previous study in the area found that two-thirds of stores had less healthy interior advertisements displayed while 98% had less healthy impulse buys [60]. As demonstrated by the robust association between the ratio of healthy-to-less-healthy food shelf-space and HEI-2010 scores, the *balance* of healthy and less healthy store features likely has a high relevance to customer behavior [32, 36, 45]. Similar to the idea of a “food swamp” [72] (which recognizes that a relative abundance of less healthy food venues in a geographic area can contribute to poor dietary outcomes among residents [73]), less healthy options inside a single store may be more likely to prevail when only a token amount of healthy options are available [21, 46, 74].

Research on interventions to promote healthful purchases in small food stores has recognized the complexity of such endeavors [47], demonstrating mixed success in changing customer outcomes [15, 17, 28, 51, 52, 75]. Beyond availability and marketing, using demand-driven and community engagement strategies have been recognized as key to successful interventions [47, 72]. More work is needed to create reasonable policies and programmatic approaches that reach stores that have not historically participated in intensive interventions. This includes working within the capacity of managers from non-traditional store types, like gas-marts, dollar stores, and pharmacies.

Indeed, store type likely plays an important role in the types of purchases that customers make. Customers may be conditioned to seek out or avoid certain store types when they are looking for a healthy snack, irrespective of the actual store environment [26, 45]. At the same time, store type and store environment features are highly correlated, often because availability and marketing are largely consistent within chain stores. Including store type in the model may, therefore, have over-controlled for relevant store characteristics.

This study has several limitations. Given its cross-sectional design, we cannot imply a causal relationship between store features and food purchasing. Second, the shelf space measures were based only on the amount of fresh fruits, vegetables, less healthy beverages, and salty snacks in the store. Undoubtedly, other healthy options that stores offered were not reflected in the measures, including frozen fruits and vegetables, whole grains, and lean proteins. We also did not obtain data on other store features that are rarely measured but may nonetheless be relevant to customer purchasing patterns, such as store cleanliness or other aesthetics [76–78].

Additionally, the participation rate of 35% in this study could introduce bias, though it is not possible for us to know if customers who participated were more or less likely to make healthier purchases. Of note, anyone exiting stores who carried a bag and did not engage with data collectors was marked as a refusal, even though they may have been ineligible, which may have resulted in an artificially low response rate. Another consideration is that the study was conducted in a relatively small geographic area and results may not be generalizable to other areas. However, in conjunction with the broader body of small food store literature, our results are consistent with patterns of purchases observed in other studies, and are unlikely to be explained by selection bias or characteristics of the particularly study setting.

This study also has a number of strengths. Few previous studies have used objective data to examine how health-promoting store characteristics are related to purchases. Furthermore, our findings are relevant in the context of the growing focus on the contribution of small food stores to the urban food environment by public health practitioners and policymakers, nationally and locally. The study may have implications for stores participating in federal food assistance programs such as Supplemental Nutrition Assistance Program (SNAP) [79], as more rigorous store stocking standards have recently been outlined by the USDA. These changes will primarily affect small and non-traditional stores, as larger grocery stores likely already meet the proposed requirements.

## Conclusion

Customer purchases of fruits and vegetables were higher in stores with the greatest amount, variety, and shelf-space of produce, and overall healthy food supply was associated with overall healthier purchases. However, in small and non-traditional food stores, healthy food marketing may be overwhelmed by ubiquitous marketing for less healthy options. Findings from this study in combination with results from previous studies suggest that efforts must be made to increase the supply and promotion of healthy items while simultaneously reducing the supply and promotion of less healthy options. New recommendations for small food stores and recent policy initiatives should be rigorously evaluated to create sustainable strategies with a wide reach to improve customer purchases, particularly in understudied businesses like gas-marts, dollar stores, and pharmacies.

## Additional file

Additional file 1: Recruitment and Sample Details. (DOCX 16 kb)
