# Supplementary material for: Association between store food environment and customer purchases in small grocery stores, gas-marts, pharmacies and dollar stores
Source: Int J Behav Nutr Phys Act. 2017 Jun 5;14:76. doi: 10.1186/s12966-017-0531-x (PMC5460502; doi:10.1186/s12966-017-0531-x)
Supplement: Additional file 1: — Recruitment and Sample Details. (DOCX 16 kb) [file 12966_2017_531_MOESM1_ESM.docx]

**Recruitment and Sample Details**

Data collectors approached everyone who was eligible for the study and exited a store in the study sample during the recruitment period. Eligible customers were ≥18 years old, spoke English well enough to respond to survey and had just made a food or beverage purchase.

A notable proportion of individuals leaving stores were not purchasing foods/beverages, particularly at food-gas marts, dollar stores, and pharmacies; they were instead purchasing items such as newspapers, gasoline, tobacco, or home goods. Thus, a visual eligibility assessment (e.g., presence of a visible food, beverage, or bag of unknown purchases) was the first step in determining eligibility. Customers were invited to participate using a brief recruitment script, verbally screened for eligibility, read an informed consent statement, and given written information about the study.

For each individual who exited the store but did not complete a survey, data collectors recorded the reason for non-participation (e.g., ineligible because under 18; ineligible because non-English

speaker; ineligible because no food, beverage, or store bag purchase (based on visual and/or

verbal assessment); or refused/no response) and the person’s apparent gender (male, female,

don’t know) and presumed race/ethnicity (White/Caucasian, Black/African American, Asian, Hispanic, don’t know). Customers who were not approached because they were clearly under age 18 were not recorded on the form. Non-English speakers were documented if a language barrier was identified after interacting with an individual. If the individual appeared to be eligible but ignored the data collector or said they were not interested, data collectors marked them as refusals. Data collectors did not record non-participants who exited the store while both data collectors were busy conducting surveys.

The gender distribution of participants was not significantly different from non-participants

(p=0.16), though the racial/ethnic distribution was significantly different (p<0.01)

Fifty-three percent of participants self-identified as White, compared to 61.3% of non

participants that were presumed to be White. In contrast, 39.8 percent of participants self

identified as Black, compared to 27.7% of non-participants. Additional details about recruitment and representativeness of sample can be found in the following manuscript: <https://www.ncbi.nlm.nih.gov/pubmed/27716142> (Pelletier et al. Successful customer intercept interview recruitment outside small and midsize urban food retailers. [*BMC Public He*alth.](https://www.ncbi.nlm.nih.gov/pubmed/27716142) 2016 Oct 5;16(1):1050.)

The recruited sample contained 668 participants. However, the analysis included only participants for which data were also available at the store-level (n=601). We also excluded one general retailer in the sample because it did not fit into our four store type (n=7 participants). Thus, the final analytic sample had 594 participants. Comparisons between the analytic sample (n=594) and cases eliminated from the recruited sample for analyses (n=74) show no significant differences (Chi-square, p<.05) by age or employment status, but the analytic sample was more likely to male, white, and college educated.

In addition to the 74 cases that we did not include in our analytic sample (see above) we had limited missing data among some measures. Cases with missing data for a particular variable in a model were not included in that model.
